# Managing Shoulder Instability in the Contact and Collision Athlete

**DOI:** 10.1007/s12178-026-10011-4

**Published:** 2026-04-28

**Authors:** Emily N. Lau, Cortez L. Brown, Rachit Saggar, Joseph Mullen, Abdulganeey Olawin, Kiera Lunn, Albert Lin, Justin J. Hicks

**Affiliations:** Department of Orthopaedic Surgery, University of Pittsburgh Medical Center, Pittsburgh, PA USA

**Keywords:** Shoulder instability, Collision athletes, Contact athletes, Glenoid bone loss, Return to play

## Abstract

**Purpose of Review:**

condition among collision and contact athletes, frequently resulting in significant time away from sport and long-term functional limitations. Due to the high physical demands and risk of recurrent injury in this population, accurate diagnosis and treatment are essential. This review provides a comprehensive overview of the clinical assessment and management of shoulder instability in these high-risk athletes.

**Recent Findings:**

Shoulder instability can be managed non-operatively or operatively. Recurrent instability is common with non-operative treatment, particularly in young male contact and collision athletes. Surgical management significantly reduces recurrence and improves return-to-sport outcomes. Anterior instability treated with arthroscopic Bankart repair generally demonstrates good results, but risk factors for failure include multiple dislocations, hyperlaxity, glenoid bone loss, and delayed intervention. Augmentation with remplissage improves outcomes over Bankart repair alone and yields results comparable to the Latarjet procedure, which is reserved for critical glenoid bone loss but is technically demanding. Posterior instability treated with arthroscopic posterior capsulolabral repair shows high return-to-sport rates and superior patient-reported outcomes compared with conservative management.

**Summary:**

Clinical decision-making should prioritize restoring stability, regaining function, and facilitating a safe return to sport. Patient-specific factors such as age, level of play, athletic goals, risk of recurrence, and extent of bone loss must also be carefully considered. A thorough understanding of shoulder instability in this population is essential to guide management and minimize the risk of recurrent instability, progressive bone loss, and long-term shoulder dysfunction.

## Introduction

Shoulder instability is a potentially debilitating injury that commonly occurs in athletes who engage in collision and contact sports due to the repetitive overhead motion of the joint and high-energy collision during play [1, 2]. This can lead to intra-articular injuries that can affect the choice and outcomes of treatment [3]. Shoulder instability is commonly seen in young individuals, with the United States Military Academy having a 1-year incidence rate of 2.8%, with 84.6% of these involving shoulder subluxations [4]. In the National Football League, the incidence of shoulder instability between 2012 and 2017 was 3.6 per 100,000 player-plays overall and 4.9 injuries per 100,000 player-plays during the preseason. Secondary defensive players accounted for most of these injuries [5]. In collegiate athletes, the National Collegiate Athletic Association Injury Surveillance Program reported 31.30 shoulder instability injuries per 100,000 athlete-exposures with an average playing time loss of 8.17 days between 2009 and 2014 [6].

Collision and contact sports can be differentiated by the amount of force involved during interaction between athletes [7]. Collision sports are those where an athlete intentionally collides with other athletes during a game with high force, while contact sports are those where, although collision may occur, the force of collision is typically lower [8]. Examples of collision sports include football, rugby, boxing, and lacrosse, where blunt trauma to the shoulder is common. Examples of contact sports include basketball, soccer, and cheerleading [8–10]. Depending on injury severity and season timeline, management of shoulder instability varies. Operative management typically ends the athlete’s season, while non-operative treatment may lead to a quicker return to play [10, 11]. Both anatomic and non-anatomic operative interventions have been developed to address shoulder instability and continue to evolve. This chapter explores effective assessment, workup, and management strategies for shoulder instability in both contact and collision athletes [12].

## Pathoanatomy

The mobility of the glenohumeral joint is achieved through a complex interplay of static and dynamic stabilizers, which, combined with the native joint anatomy, enable the extensive range of motion essential for athletic performance. The shallow glenoid fossa and incongruent bony anatomy of the humeral head predispose the joint to instability events under high-energy forces typical of contact and collision sports [13, 14].

Static stabilizers, including the glenoid labrum and capsule-labral complex, are fundamental to shoulder stability. The labrum increases socket depth by 50%, enhancing stability through a suction-cup effect [15, 16]. The inferior glenohumeral ligament (IGHL) serves as the primary restraint against anteroinferior translation, particularly during abduction and external rotation, positions frequently assumed in high-impact tackling and overhead blocking [17]. These static components are complemented by dynamic stabilizers, including the rotator cuff and periscapular musculature, which provide critical force-coupling and compression effects for maintaining glenohumeral joint congruence [18, 19].

Understanding pathoanatomic mechanisms is fundamental to managing shoulder instability in contact and collision athletes. Video analyses have identified four primary mechanisms in rugby: the “try scorer,” involving hyperflexion of the outstretched arm causing anterior instability and associated rotator cuff injuries; the “tackler,” resulting in anteroinferior instability through abduction and external rotation forces; the “direct impact,” leading to posterior instability and glenoid bone loss; and the “poach position,” which stresses the capsule-labral complex through combined flexion and internal rotation [20, 21]. In rugby, tackling accounts for 77% of shoulder injuries [22]. American football demonstrates position-specific patterns, with offensive linemen experiencing unique posterior instability patterns due to repetitive blocking mechanics, while defensive backs show higher rates of anterior instability with associated osseous injuries during high-velocity tackling [23, 24]. 36% of injuries were found to occur in the final quarter, and non-ball carriers, comprising 85% of cases, were found to be injured later than ball carriers. This suggests that tiredness and fatigue may play a role in injury occurrence [21].

### Anterior Instability

The pathoanatomy of anterior instability frequently extends beyond isolated lesions in contact and collision athletes. While the classic Bankart lesion (avulsion of the anteroinferior labrum) is common, contact athletes often demonstrate more complex variants, including Hill-Sachs lesions and anterior labroligamentous periosteal sleeve avulsion (ALPSA) lesions, which require specific surgical considerations due to altered biomechanics and increased risk of recurrent instability [25]. The PIT (Pittsburgh Instability Tool) has been shown to be a reliable tool to identify patients who may benefit from additional remplissage augmentation in arthroscopic cases of Bankart repair for recurrent instability (Table 2) [26]. In addition, Di Giacomo’s Glenoid Track Instability Management Score (GTIMS) provides a predictive framework for recurrence after arthroscopic Bankart repairs and aids in determining the need for soft tissue repair versus bony augmentation procedures [27, 28].

Glenoid bone loss, with critical thresholds as low as 13.5%, exacerbates instability risk, with each subsequent dislocation contributing to additional bone loss [29–31]. Bipolar bone loss must be assessed in cases of instability. The glenoid track concept encompasses the combined effects of glenoid and humeral bone loss in cases of anterior shoulder instability, as first described by Yamamoto et al. [32]. “On track” shoulders, where Hill-Sachs lesions are smaller than the glenoid articular track and less likely to engage, are often treated successfully with arthroscopic labral repair. In “off-track” shoulders with larger Hill-Sachs lesions that can lead to engagement with the glenoid rim, one may consider remplissage or bony augmentation in addition to labral repair [33]. Distance to dislocation (DTD) was introduced by Li et al. as a way to determine how close “on-track shoulders” were to becoming “off-track” and aid in identification of high-risk individuals [34]. A DTD < 8 mm was determined as the critical threshold for a significantly higher risk of failure in “on-track” shoulders treated with sole arthroscopic Bankart repair. Such shoulders were deemed to be “near-track,” and Barrow et al. further demonstrated that as DTD decreased, post-surgical rates of recurrent dislocation significantly increased [33]. Other lesions to evaluate for during workup include Glenolabral Articular Disruption (GLAD) with articular cartilage involvement and combined capsulolabral and Humeral Avulsion of Glenohumeral Ligament (HAGL) injuries.

Specific mechanisms of anterior instability in contact sports have been identified. The “try scorer” mechanism, involving hyperflexion of the outstretched arm, frequently results in anterior instability and is often associated with rotator cuff injuries. The “tackler” mechanism, involving abduction and external rotation during high-velocity collisions, is another common cause of anteroinferior instability, particularly in defensive backs and rugby players [20, 21, 23].

### Posterior Instability

Contact athletes demonstrate distinct posterior instability patterns that often demonstrate progressive changes [35, 36]. Studies have shown the posterior band of IGHL has approximately 30% less load to failure compared to the anterior band [37]. The reverse Bankart lesion, involving posterior labral avulsion in the 6 to 10 o’clock glenoid position, results in an increase of 86% posterior translation in the jerk position and an increase of 31% inferior translation in the sulcus position, with concomitant pathologies leading to further increases [38]. Progressive changes include cumulative microtrauma leading to capsular stretching, Kim lesions (posterior labral-chondral disruption), and progressive posterior glenoid bone loss [39, 40]. Instability may result from direct posterior impact. The “direct impact” mechanism, often seen in rugby and American football, involves forceful posterior loading that can lead to glenoid bone loss and reverse Bankart lesions. Offensive linemen are particularly susceptible due to repetitive posterior-directed forces during blocking maneuvers [21, 23].

### Multidirectional Instability

Multidirectional Instability (MDI) in contact athletes presents unique pathoanatomic features affecting both capsular integrity and neuromuscular function. Studies have shown a global capsular volume increase of 15–25% compared to normal shoulders, with a critical 20% threshold for symptomatic instability. These changes are accompanied by altered collagen composition in those with recurrent instability [41]. Neuromuscular dysfunction manifests as delayed rotator cuff activation and decreased force coupling efficiency, with EMG studies confirming altered muscle firing patterns [42, 43]. Fatigue may contribute to the development of multidirectional instability. In rugby, 36% of shoulder injuries occurred in the final quarter of play, with non-ball carriers affected in 85% of cases. This supports the role of neuromuscular fatigue in increasing susceptibility to instability episodes during late-game scenarios [21].

## History and Physical Exam

A detailed history from the athlete and physical exam are critical to determine a diagnosis. When obtaining an injury history, one should query about the following: mechanism of injury, onset of symptoms, quantity of instability events, reproducibility of instability, joint laxity, and prior shoulder injuries. A shoulder exam should be completed in a systematic fashion. This includes inspection, palpation, active and passive range of motion, and provocative maneuvers. Several provocative physical exam maneuvers have been shown to identify shoulder instability with high accuracy [44].

**Fig. 1:**
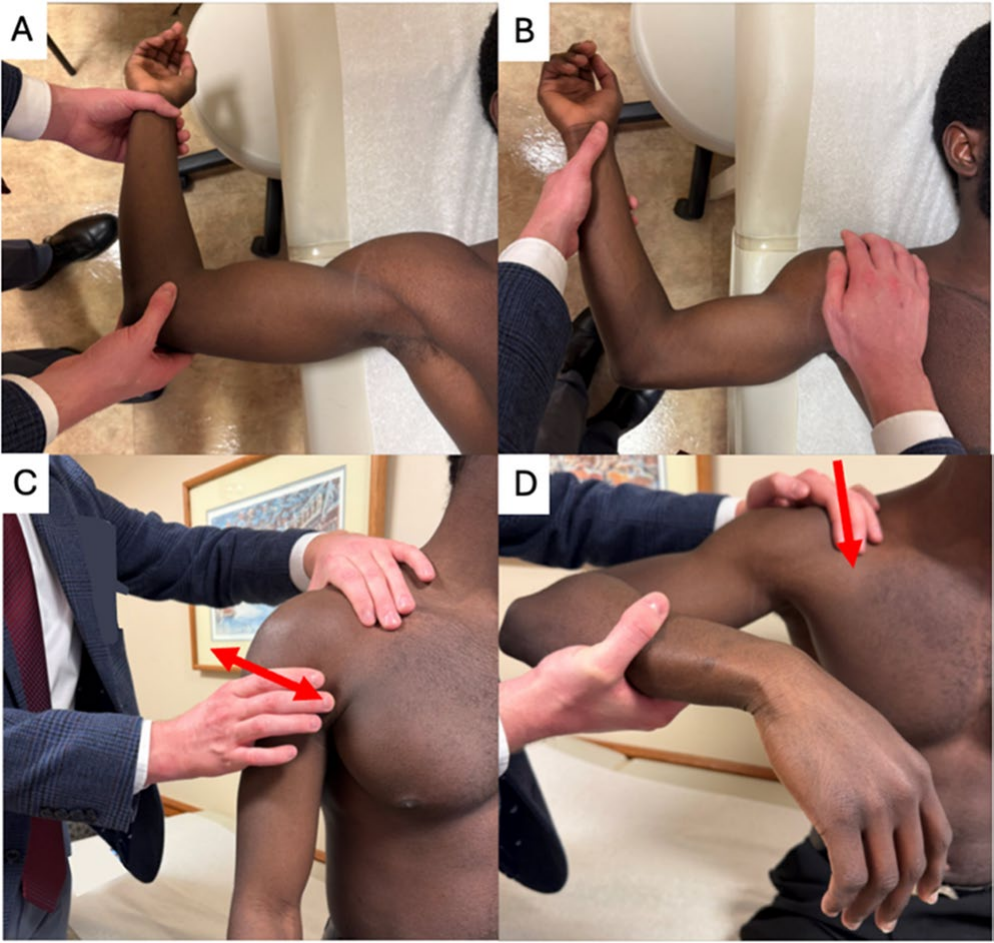
Provocative maneuvers to assess anterior shoulder instability. (**a**) Anterior apprehension test; (**b**) Jobe relocation which is done directly after a positive anterior apprehension test; (**c**) Anterior load and shift test; and (**d**) Gagey test

The following provocative maneuvers are useful for assessing anterior instability [44]: anterior apprehension, Jobe relocation, surprise, and anterior load and shift tests. The anterior apprehension test can be performed standing or supine. The injured shoulder is abducted to 90° and maximum external rotation. An anterior force is applied to the posterior humeral head. The patient endorsing apprehension, not pain, results in a positive test (Fig. 1) [45]. To reduce apprehension, a posterior force is applied to the anterior humeral head. This is coined the Jobe relocation test. The surprise test involves a sudden removal of posterior force to the humeral head. If the patient endorses recurrent apprehension, this is considered a positive test. Finally, the load and shift test can be performed standing or supine. While one hand is stabilizing the shoulder girdle, the other is cupping the humeral head and attempting to translate it anteriorly and posteriorly. Instability can be graded using the Modified Hawkins scale (Table 1) [44].

**Table 1:** Modified Hawkins scale for anterior shoulder instability

Grade	Description
**Grade 0**	No translation
**Grade 1**	Translation up to the glenoid rim without subluxation
**Grade 2**	Translation over the rim with spontaneous reduction
**Grade 3**	Translation over the rim without reduction (dislocation)

**Fig. 2:**
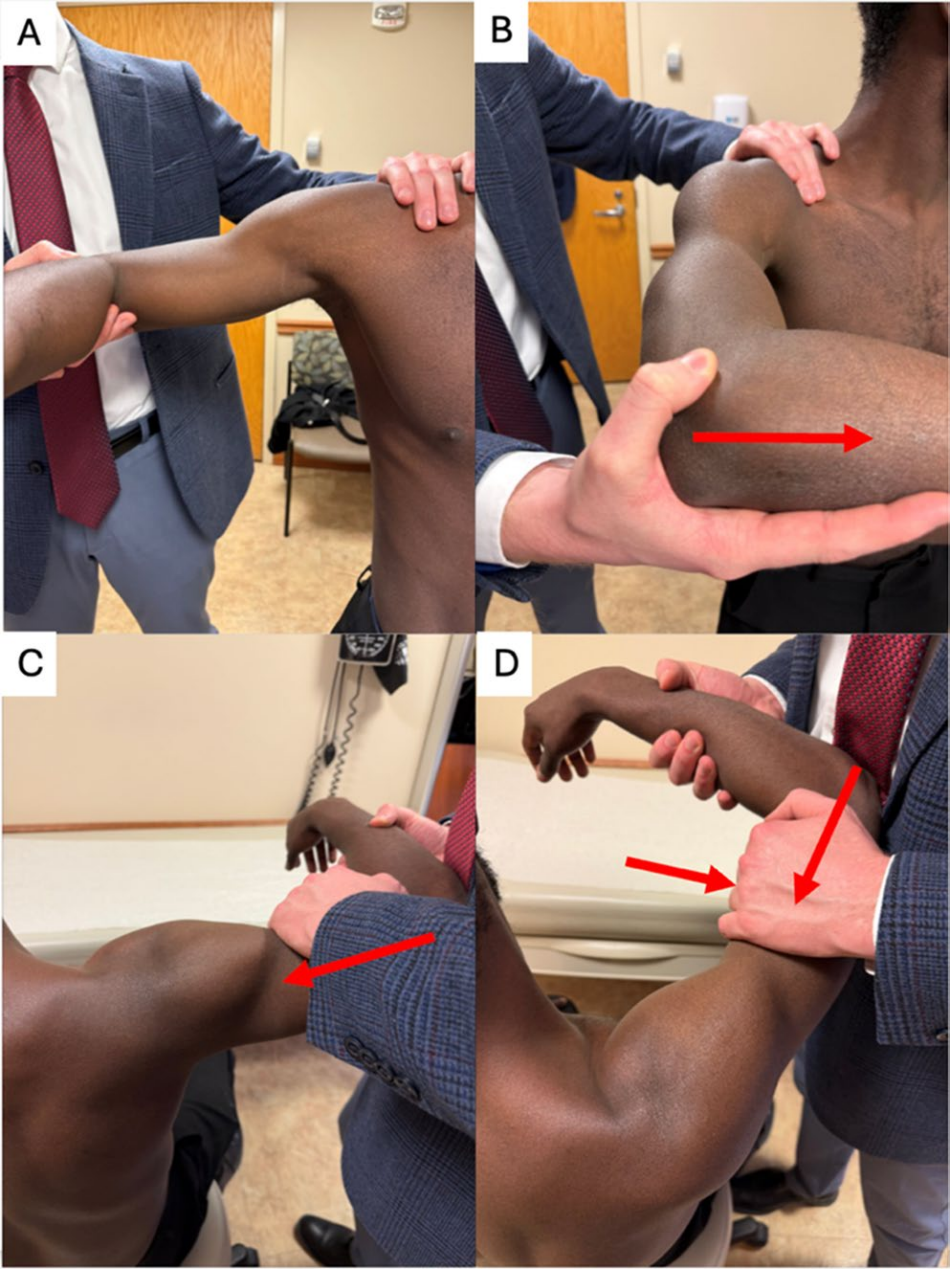
Posterior Instability Provocative Tests Positioning. (**a**) Jerk Test Setup: The examiner stabilizes the scapula while holding the elbow, positioning the shoulder in 90° flexion and internal rotation. (**b**) Jerk Test Final Position: An axial load is applied while horizontally adducting the arm. A posterior “jerk” or clunk indicates posterior instability. (**c**) Kim’s Test Setup: The patient is seated, and the examiner supports the elbow and proximal humerus with the shoulder in 90° abduction. (**d**) Kim’s Test Final Position: An axial load and 45° upward force are applied while pushing the elbow posteriorly. Pain or instability suggests a posterior labral tear

The Jerk and Kim tests are useful in assessing posterior instability (Fig. 2) [46]. To perform the Jerk test, the patient is seated while the examiner is behind them. While stabilizing the scapula with one hand, the examiner abducts and internally rotates the injured shoulder to 90°. Next, an axial force is applied in the horizontal plane through the humerus, while simultaneously adducting the arm. A positive test occurs if pain or a clunk is elicited. The Kim test [47], which assesses for posteroinferior labral pathology, can also be performed seated. The examiner will abduct the patient’s shoulder and flex their elbow to 90°. One hand will cup the proximal forearm while the other will cup the lateral part of the proximal arm. An axial force is applied to the humerus along with shoulder adduction and an upward elevation 45° toward the patient’s face. Posterior load and shift testing is performed as described in the anterior load and shift test, but a posterior force is applied across the glenohumeral joint.

General ligamentous laxity should be assessed in all patients using the Beighton Score, with scores of 5 or more indicating joint hypermobility. Hyper-external rotation with the arm at the side or a load and shift test score of 2 + or more in two or more planes, should raise suspicion for multidirectional instability or baseline laxity.

For inferior instability, the Hyperabduction (Gagey) test offers moderate sensitivity and specificity [48]. With the patient supine, the examiner will apply a downward force about the shoulder girdle while abducting, with the elbow flexed at 90°. Apprehension noted at 105° or more of shoulder abduction is considered a positive test.

In contact and collision athletes, there should be a high suspicion for concomitant pathology in the shoulder. The onus is on the examiner to be comprehensive in their examination and clinical workup and develop an appropriate diagnosis.

## Clinical Work Up

### Imaging

**Fig. 3:**
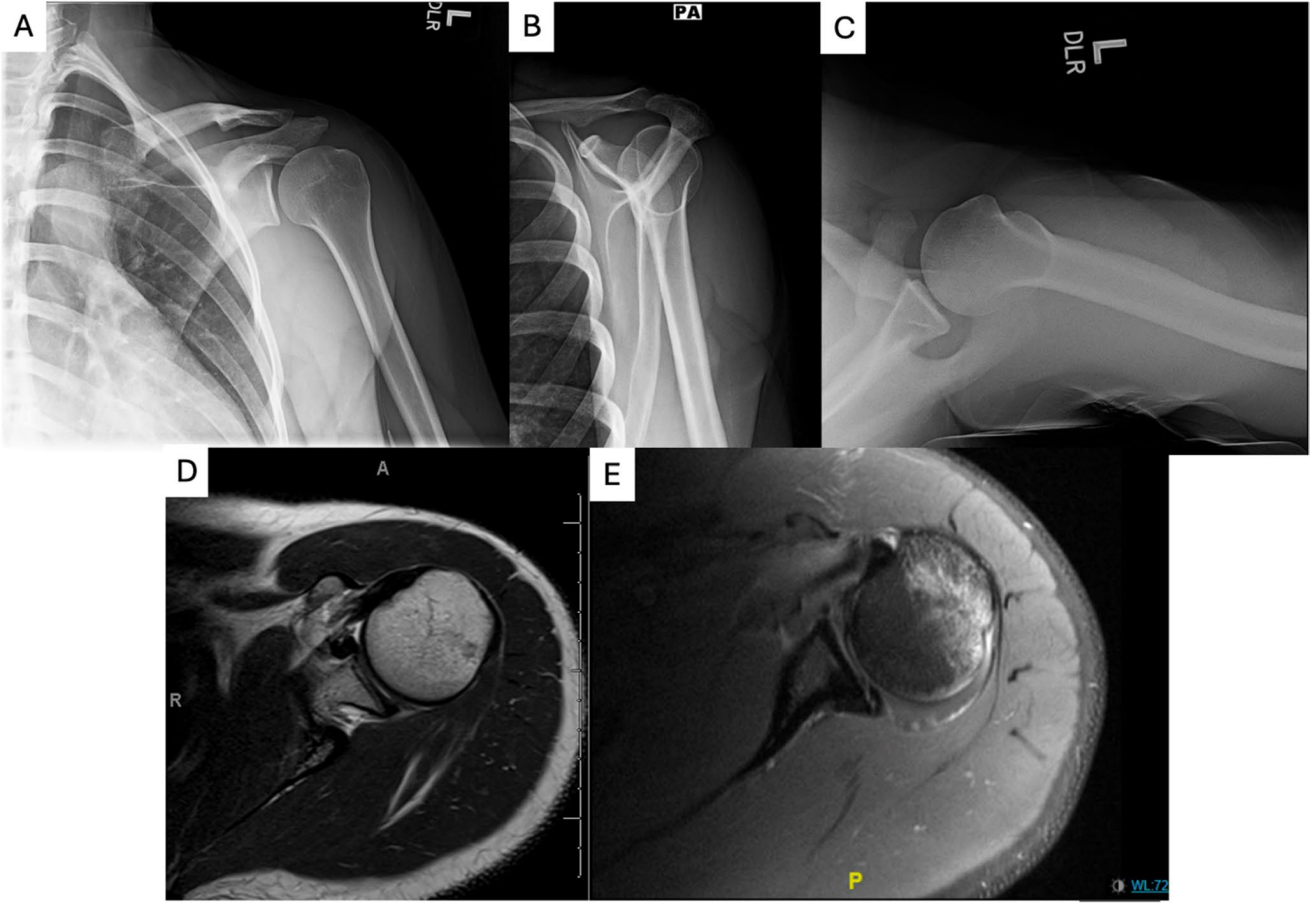
Diagnostic Imaging for Shoulder Instability. (**a**) AP Oblique View on X-ray to assess glenohumeral alignment and bony defects; (**b**) Scapular Y View on X-ray to evaluate humeral head positioning and acromion morphology; (**c**) Axillary Lateral View on X-ray to detect posterior dislocation and glenoid bone loss; (**d**) Axial View on MRI highlighting anterior labral tear; and (**e**) Axial View on MRI highlighting a Hill-Sachs lesion

Plain radiographs of the shoulder should be obtained after injury, including anteroposterior, Grashey, axillary lateral, and scapular Y views (Fig. 3) [49]. Previous evidence has shown accurate detection of glenoid and humeral defects [50, 51]. Specific views for glenoid bone loss include the Bernageau, West Point, and Dideé views [52]. Regardless of anterior or posterior instability, a 3D computed tomography (CT) scan is useful for evaluation of fractures, glenoid bone loss (GBL), humeral defects, and Hill-Sachs lesions (HSLs) [53–59]. However, clearly defined thresholds for GBL have only been established for anterior glenohumeral instability at this time [60].

When evaluating the soft tissue structures of the shoulder joint, magnetic resonance imaging (MRI) should be obtained. This modality allows for complete evaluation of the labrum and rotator cuff in addition to HSLs [61]. MRI arthrogram (MRA), which utilizes an intra-articular injection of gadolinium, may be more sensitive in identifying pathology in patients with capsular insufficiency [61]. However, given the improvement in MRI quality, arthrograms do not need to be routinely ordered as a meta-analysis identified only a “marginal” difference [62] in accuracy [61]. Cong et al. demonstrated that if MRI is performed less than two weeks from an acute anterior instability event, MRI is equivalent to arthrogram in identifying pathology [63]. In addition, this study demonstrated diminished accuracy and precision over time, in identification of labral tears status post dislocation. This underscores that selection of imaging must be done on a case by case basis.

Imaging the shoulder while in abduction and external rotation (ABER) improves MRI accuracy evaluation of the anterior band of the inferior glenohumeral ligament (AIGHL) [64]. As previously discussed, it has been demonstrated that the risk of recurrent dislocation in on-track” shoulder lesions, increases significantly as the “distance to dislocation” (DTD) approaches 0 (i.e. a near “off-track” lesion) [34]. As such, this calculation should be considered when planning for arthroscopic Bankart repairs [33].

## Management

Management of shoulder instability in the contact and collision athlete is centered around (1) restoring stability, (2) regaining function and (3) returning to sport.

### Anterior Shoulder Instability

**Fig. 4:**
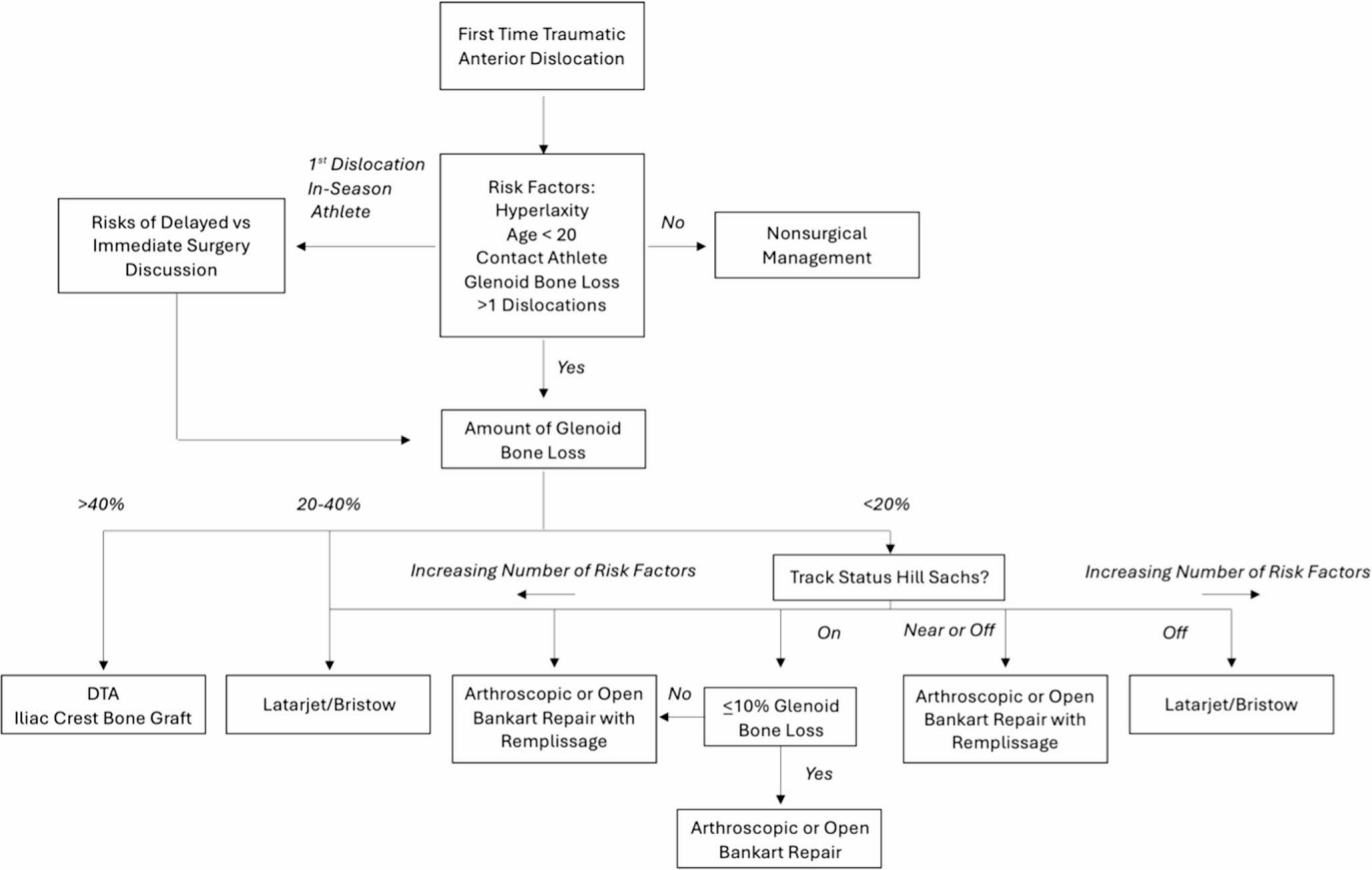
Anterior Instability Management Decision-Tree for Athletes: Non-operative and Operative Options

Generally, anterior shoulder instability is not well tolerated in contact athletes. Prior to returning to play, restoration of motion and strength is imperative (Fig. 4) [65].

### Non-Surgical Treatment

A variety of non-surgical treatment protocols exist, but generally, athletes begin with a brief period of immobilization ranging from 3 to 7 days. Thereafter, they progress to early physical therapy centered around regaining range of motion and strengthening the rotator cuff and surrounding musculature. As strength returns to baseline and is comparable to the contralateral shoulder, athletes can move forward with sport-specific activity in a gradual fashion [66–70]. After demonstrating symmetric strength and pain-free range of motion without clinical apprehension, athletes may return to sport. Sport activity can be completed with or without the brace. A player’s psychological readiness should also be considered and can dictate success in return to sport regardless of operative or non-operative treatment regimen [71].

There are varying opinions on the timeline and position of shoulder immobilization. This can be further complicated by athletes that suffer in-season injuries and desire to continue to play. There is some evidence to support that anterior labral structures are better reduced anatomically with the shoulder in external rotation. A recent systematic review of over 800 patients found a 44% reduction in instability recurrence risk when patients were immobilized in external rotation compared to internal rotation [11]. Many of the existing non-operative protocols do call for bracing; however, no studies have demonstrated an explicit decrease in recurrent instability with brace use. Certainly, functional bracing can limit extremes of range of motion where athletes are at risk of instability episodes. This may come at a cost of players being able to execute specific sport maneuvers, particularly overhead motions [66, 67, 72].

The management of the in-season athlete sustaining a primary anterior instability event remains controversial, and decisions are made on an individual basis, considering age, type and level of sport, position, future athletic goals, and anatomic factors including bipolar bone loss. If an athlete desires to defer surgical intervention and return to in-season play, a thorough discussion with the athlete and, as necessary, the athlete’s guardian is imperative. This discussion should detail risk of recurrence, what sport activity can realistically be performed with the remaining in-season time, and also detail the risk of glenoid bone loss, cartilage injury, and further labral tearing [73–75]. The increased severity of concomitant injuries which occurs with each subsequent instability event also has important ramifications regarding the treatment approach and the success of future surgical stabilization. It is important to note that the outcomes for nonoperative treatment following in-season anterior instability events are not synonymous for return to play and recurrence of instability. While the literature has shown high rates of return to sport within the same season, few athletes will complete the season without another instability event [66, 76, 77]. During this time, the goal is to mitigate recurrent instability events until the off-season when athletes can rest more extensively or potentially undergo operative intervention.

In-season return to play may be contraindicated in patients with extensive bone loss. The literature describes glenoid bone loss > 20% as a contraindication for a contact athlete returning to play. Other relative contraindications for return include large or “off track” Hill-Sachs lesions, humeral avulsion of the glenohumeral ligament, and concomitant rotator cuff tear.

As mentioned, recurrent instability is not uncommon. This is particularly true in young male contact and collision athletes, with several studies demonstrating a failure rate over 70% in athletes younger than 18 years of age.

### Surgical Treatment

Given the daily impact and trauma that occurs within the contact and collision athlete population, coupled with the high rates of failure with non-operative treatment, surgical intervention is often pursued.

Multiple randomized controlled trials have shown a significant reduction in instability recurrence rates after operative intervention compared to nonoperative treatment, with rates of 10% and 70%, respectively. Even after a first-time dislocation, surgical intervention is recommended due to recurrence rates as high as 58% at long-term follow-up [78]. Furthermore, subsequent instability events, even 1 further dislocation after a 1st time dislocation, can cause further damage and worse clinical outcomes. Decreased failure rates were seen in patients undergoing early arthroscopic Bankart repair following a primary dislocation compared to those undergoing surgical repair after a second dislocation [79]. Arthroscopic stabilization has also been shown to result in **i**mproved event-free survival and higher rates of return to sport when compared to those being managed nonoperatively, and this difference was exaggerated further within the cohort of athletes playing high-risk sports [28, 80–82]. The risks of delayed surgical treatment should be compared to the importance of continuing in-season participation.

Indications for immediate surgical management following primary instability events in the contact and collision athlete include those with anatomic risk factors for recurrence, including acute glenoid rim fractures (“Bony Bankart”) and bipolar bone loss [83, 84]. Additionally, surgical intervention is recommended for patients with recurrent instability events after attempting nonoperative management. Several procedures exist depending on patient presentation and underlying pathology.

#### Arthroscopic Bankart Repair

In cases of anterior shoulder instability, arthroscopic Bankart repair is commonly performed and has demonstrated successful outcomes. Recurrent instability rates are cited between 4 and 19%, and athletes demonstrate high rates of return to play [77, 84]. Bankart repair utilizing four anchors with deliberate spacing within the defect is recommended to minimize risk of failure. Attention should be given to the trajectory of the anchor to best replicate the anatomy of the glenoid and labrum [85–87]. Other operative technical recommendations are as follows: including an additional anterosuperior portal to optimize visualization of the anterior glenoid, avoiding anchor placement superior to the 3 o’clock position, incorporating capsular plication into labral stitches to mitigate capsular volume distention, and using posteroinferior capsular stitches to balance the reduction of the glenohumeral joint.

Despite good outcomes reported in the literature, it is important to identify risk factors for recurrence following arthroscopic Bankart repair. These include multiple previous dislocation events, hyperlaxity, glenoid bone loss (GBL), presence of an “off-track” or “near-track” lesion, and longer time lapse from injury to surgical intervention. The Instability Severity Index Score (ISIS) and the Glenoid Track Instability Management Score (GTIMS) can aid in calculation of the risk of failure of arthroscopic Bankart repair alone. These tools can aid in the decision of performing adjuncts to Bankart repair or if proceeding with a bony restoration procedure may be necessary [86, 87]. The PIT score can also aid in decision-making and surgical planning (Table 2) [26]. Arthroscopic Bankart alone has been associated with 30% failure rates at 10-year follow-up, which has contributed to the increased use of augmentation with remplissage [88]. In higher risk cases, it is imperative to have a detailed discussion with the patient regarding elevated risk of recurrence with delayed arthroscopic management or possible need for more aggressive, open approaches to mitigate recurrence.

**Table 2:** PIT (Pittsburgh instability Tool) scoring Criteria. This tool was subsequently used to risk-stratify patients into the following subgroups: low-risk (0–3), moderate-risk (4–8), high-risk (9–13), extreme-risk (14+) with regard to recurrent instability

*Prognostic Factors*	*Points*
**Patient Age at Surgery** *Under 17 years* *17–19 years* *20–24 years* *25 + years*	6 4 2 0
**Contact Sport Status** *Contact Sport Athlete* *No Contact Sports (Evaluate GBL)* * GBL < 5%* * GBL 5–10%* * GBL 11–15%* * GBL 16–20%*	7 See below: 0 1 2 3
**Shoulder Laxity Evaluation Under Anesthesia** *Hyperlaxity* *No evidence of hyperlaxity*	5 0
**Distance to Dislocation (mm)** *Near-Track Lesion (DTD </= 10 mm)* *On-Track Lesion (DTD > 10 mm)*	4 0
**Preoperative Instability Episodes** *2 + Instability Events* *Only 1 Instability Event*	3 0
**Primary Arthroscopic Stabilization** *Bankart Repair Only* *Bankart Repair + Remplissage*	0 −8

#### Remplissage

**Fig. 5:**
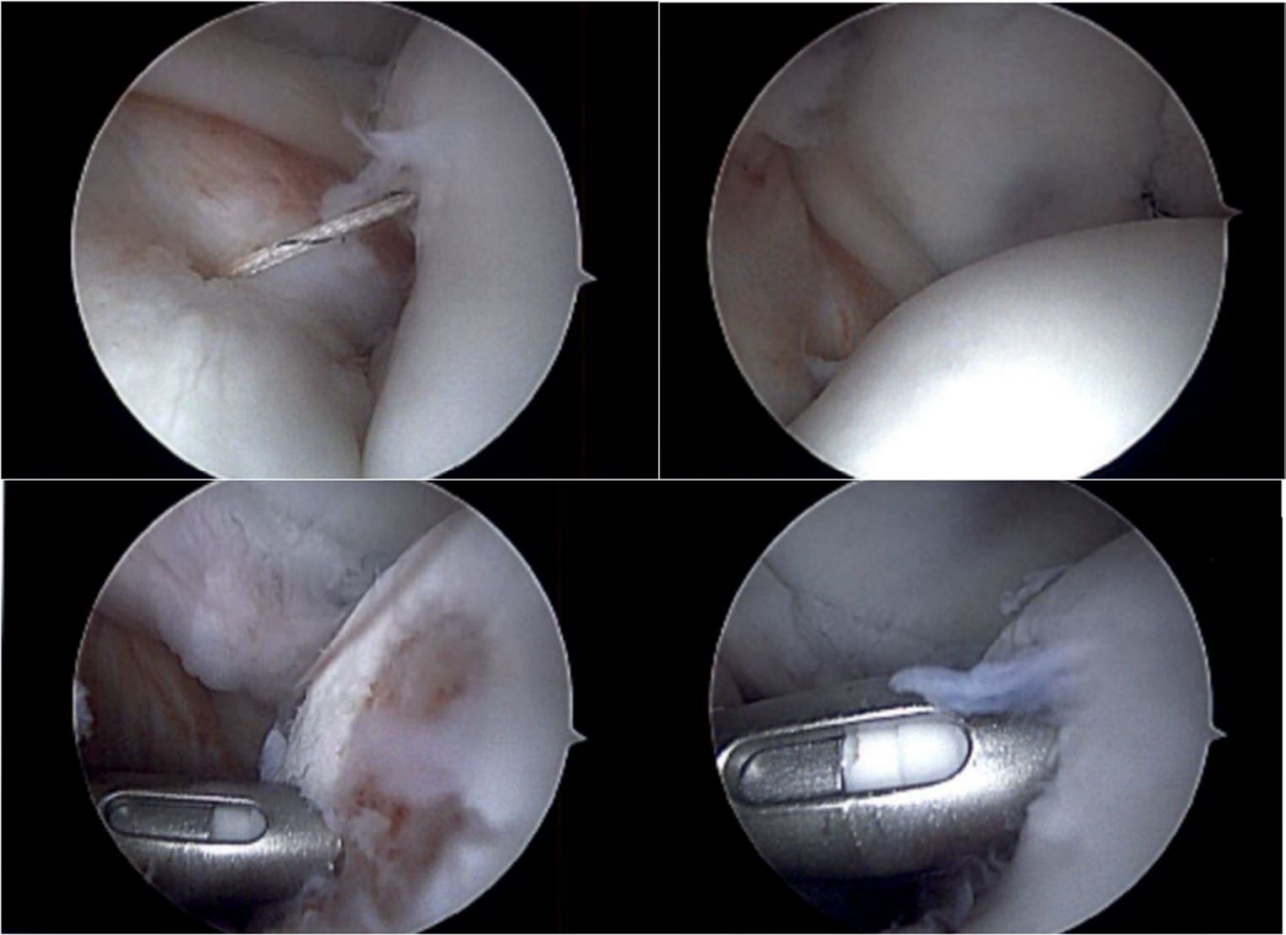
Intraoperative Arthroscopic Images of Remplissage Procedure

Remplissage secures the infraspinatus tendon and associated joint capsule into the Hill-Sachs defect (Fig. 5). In turn, this creates an extra-articular defect and mitigates risk of further engagement between the defect and glenoid. In patients with “off-track” Hill-Sachs defects and GBL less than 25%, remplissage has been effective in restoring stability to the glenohumeral joint. As mentioned above, isolated arthroscopic Bankart repair alone has been associated with high failure rates at long term follow up, which has generated substantial interest in remplissage [88]. A recent randomized control trial (RCT) by MacDonald et al. demonstrated that addition of remplissage decreased recurrent instability, and multiple systematic reviews have demonstrated lower rates of recurrence, improved return to play and decreased revision rates with the addition of remplissage for both on- and off-track lesions [89–92]. Other indications include contact and collision athletes with “near-track” lesions and those who have failed primary Bankart repair, even without critical bone loss. Instability recurrence rates have been reported as low as 5% throughout the literature. In contact and collision athletes with “on-track” or “near-track” lesions and subcritical bone loss, remplissage should be strongly recommended given the risk factor burden and increased failure rate with Bankart repair alone [9]. Studies have also demonstrated equivalent outcomes for arthroscopic Bankart with remplissage when compared to Latarjet in cases of subcritical bone loss [93]. Although some studies have cited decrease in external rotation after remplissage, recent systematic reviews have not demonstrated as such [83, 92, 94, 95].

Operative technical recommendations are as follows: including the use of two anchors in the humeral head defect and obtaining direct visualization of sutures being tied to ensure proper reduction of the tissue into the defect, whether intra-articularly or in the subacromial space. The addition of humeral head bone grafting may be considered in cases of larger Hill-Sachs defects [83].

#### Additional Soft Tissue Procedures

Dynamic anterior stabilization (DAS) and arthroscopic subscapularis augmentation (ASA) are newer procedures to address shoulder instability. DAS utilizes the long head of the biceps tendon to augment the anteroinferior glenoid, while ASA utilizes the upper third of the subscapularis to augment the anteroinferior glenoid. Further studies looking at clinical outcomes and efficacy of these procedures are needed to fully understand their effect and role for utilization in the contact or collision athlete.

#### Coracoid Transfers

**Fig. 6:**
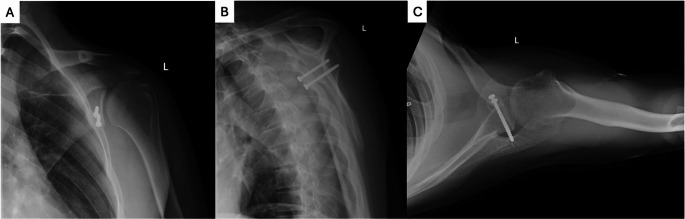
Post-operative Radiograph Views of Latarjet Patient: (a) AP Oblique View; (b) Scapular Y View; and (c) Axillary Lateral View

The Bristow and Latarjet procedures both function to restore glenoid bone stock and provide further soft tissue stability about the shoulder. The Latarjet involves transfer and fixation of the tip of the coracoid to the anterior glenoid (Fig. 6). This not only restores bone loss, but promotes stability via the sling effect of the conjoint tendon along the anteroinferior glenoid. The capsule can subsequently be repaired to the stump of the coracoacromial ligament to further resist anterior translation [96, 97]. Latarjet is often utilized for patients with recurrent instability and critical GBL with instability recurrence rates reported as low as 0.8% [83, 98, 99]. Previously, GBL > 20% was used as the critical bone loss threshold; however, some are now recommending Latarjet with any GBL > 13.5%. Utilizing verified tools such as the PIT score, history and physical exam can be extremely useful to determine whether someone would benefit from arthroscopic Bankart with remplissage or require another approach with glenoid bone loss < 20%.

Despite good outcomes reported in the literature, the Latarjet can be technically demanding, and complication rates have been reported as high as 30%, including neurovascular injury, failure of fixation, graft nonunion, graft resorption, development of osteoarthritis, and postoperative stiffness [100]. As mentioned above, concomitant remplissage and arthroscopic Bankart repair in the setting of subcritical GBL has been shown to have similar recurrent instability rates to Latarjet [93].

### Posterior Shoulder Instability

#### Non-surgical Treatment

In cases of recurrent posterior subluxation, the mainstay of treatment is physical therapy. Rehabilitation is centered around improving proprioception, strengthening the rotator cuff and surrounding periscapular musculature. If athletes experience persistent pain or instability or are unable to return to sport despite a course of physical therapy, surgical intervention can be considered.

#### Surgical Treatment

Surgical intervention involves capsulolabral repair. Compared to anterior shoulder instability, less evidence exists with regards to timing of operative intervention and when consideration should be given to bony augmentation. In cases of posterior instability, higher GBL did correlate with higher nonoperative failure rates, with 15% bone loss showing a 25 times higher failure rate [101]. Nonetheless, arthroscopic posterior capsulolabral repair demonstrates high rates of return to play and high patient-reported outcomes compared to athletes treated without surgery [102]. Revision rates were low, cited at 5.4% at a minimum 4 year follow up [103]. Patients with smaller glenoid bone widths and higher degrees of preoperative instability may be a risk for failure of repair. Future studies are required to determine when bony augmentation could benefit patients. At this juncture, cases with GBL > 20%, significant humeral head retroversion, and laxity of the soft tissues in the posterior shoulder should pique providers’ consideration for bony augmentation. Open procedures may be necessary in cases of retroversion or failed initial repair. Reverse Hill-Sachs lesions may require disimpaction, bone grafting or tenodesis of the subscapularis.

### Postoperative Rehabilitation Protocol

The post-operative rehabilitation process following anterior glenohumeral stabilization procedures often involves three phases: immobilization, passive and active-assisted exercises, and active motion and strengthening exercises with sport-specific activities. Following arthroscopic Bankart repair, 3 to 6 weeks of sling immobilization allows for provisional healing of the capsuloflabral repair. Following Latarjet, a similar immobilization is implemented. Thereafter, patients begin passive and active-assisted exercises for 4 to 6 weeks before progressing to a strengthening program.

Return to sport typically occurs after 4 to 6 months, but can vary depending on an individual athlete’s activity and goals. Return to sport testing has been shown to be effective in identifying residual strength or functional deficits, which may compromise an athlete’s safe return to sport without setback or repeat injury. In a study of 43 athletes, the use of criteria-based return to sport testing 6 months after undergoing arthroscopic Bankart repair found residual strength or functional deficits in 38 of the patients [99]. Criteria-based return to sport protocol evaluating isokinetic, isometric, and functional testing of an athlete has shown excellent reliability and validity. Utilizing a criteria-based protocol has shown to reduce instability recurrence rates compared to a time-based protocol after arthroscopic Bankart repair [99, 104].

## Conclusion

Shoulder instability is a common and challenging problem in the contact and collision athlete population. This review outlines the pathoanatomy of shoulder instability, physical exam assessment, both operative and non-operative treatment options, and postoperative rehabilitation to optimize recovery and return to sport. Management of shoulder instability demands an individualized approach. Overall, early surgical intervention in high-risk contact and collision athletes, particularly those with significant bone loss or recurrent instability, often yields superior outcomes. Surgical decision making, including arthroscopic versus open approaches, should consider demographic and anatomic risk factors, including bipolar bone loss. A multidisciplinary effort is imperative to return athletes to play in optimal and timely fashion.

## Data Availability

No datasets were generated or analysed during the current study.

## KEY REFERENCES

- Charles S, Marcaccio S, Lin RT, et al. The Pittsburgh Instability Tool Score Predicts Outcomes After Arthroscopic Anterior Shoulder Stabilization in Patients With Subcritical Bone Loss. *Arthroscopy: The Journal of Arthroscopic & Related Surgery*. Published online May 23, 2025. doi:10.1016/j.arthro.2025.04.023.
  - ○ This reference was included because it introduces a reliable tool for predicting outcomes after arthroscopic anterior stabilization in patients with subcritical bone loss. It is clinically relevant for identifying patients who may benefit from arthroscopic Bankart repair with remplissage, aiding surgical decision-making and patient counseling.
- Pouges C, Hardy A, Vervoort T, Amouyel T, Duriez P, Lalanne C, et al. Arthroscopic Bankart Repair Versus Immobilization for First Episode of Anterior Shoulder Dislocation Before the Age of 25: A Randomized Controlled Trial. Am. J. Sports Med. 2021;49:1166–1174; 10.1177/0363546521996381.
  - ○ This randomized controlled trial shows that early operative intervention in young patients significantly reduces recurrent instability and improves functional outcomes compared to nonoperative management. It supports the recommendation for surgical management of shoulder instability in high-risk patients.
- Charles SJ, Marcaccio S, Herman ZJ, Steuer F, Reddy RP, Kane G, et al. Arthroscopic Bankart repair with remplissage yields similar outcomes to open Latarjet for primary and revision stabilization in the setting of subcritical glenoid bone loss. J. Shoulder Elbow Surg. 2024;33:2805–2818; 10.1016/j.jse.2024.05.003.
  - ○ This study is important because it shows that arthroscopic Bankart repair with remplissage provides outcomes comparable to the Latarjet procedure in patients with subcritical glenoid bone loss. It highlights that both techniques are effective and that the choice should be tailored to patient-specific factors and functional demands.
- Ahmed AF, Polisetty TS, Wang C, et al. Higher return to sport and lower revision rates when performing arthroscopic Bankart repair with remplissage for anterior shoulder instability with a Hill-Sachs lesion: a meta-analysis. *J Shoulder Elbow Surg*. 2024;33(8):1836–1846. doi:10.1016/j.jse.2024.01.045.
  - ○ This meta-analysis demonstrates that adding remplissage to arthroscopic Bankart repair results in higher return-to-sport rates and lower postoperative instability and revision rates in patients with Hill-Sachs lesions. It reinforces the value of remplissage as an augmentation technique in appropriate cases.
